# Application of Artificial Intelligence in Mathematical Modeling and Numerical Investigation of Transport Processes in Electromembrane Systems

**DOI:** 10.3390/membranes16010041

**Published:** 2026-01-12

**Authors:** Ekaterina Kazakovtseva, Evgenia Kirillova, Anna Kovalenko, Mahamet Urtenov

**Affiliations:** 1 Faculty of Computer Technologies and Applied Mathematics, Kuban State University, 350040 Krasnodar, Russia; kadii@fpm.kubsu.ru (E.K.); kovalenko.a@fpm.kubsu.ru (A.K.); aicenter@kubsu.ru (M.U.); 2 Faculty of Architecture and Civil Engineering, RheinMain University of Applied Sciences, Kurt-Schumacher-Ring 18, 65197 Wiesbaden, Germany

**Keywords:** electromembrane systems, neural networks, training set, numerical study, electroconvection

## Abstract

To enhance desalination efficiency and reduce experimental costs, the development of advanced mathematical models for EMS is essential. In this study, we propose a novel hybrid approach that integrates neural networks with high-accuracy numerical simulations of electroconvection. Based on dimensionless similarity criteria (Reynolds, Péclet numbers, etc.), we establish functional relationships between critical parameters, such as the dimensionless electroconvective vortex diameter and the plateau length of current–voltage curves. Training datasets were generated through extensive numerical experiments using our in-house developed mathematical model, while multilayer feedforward neural networks with backpropagation optimization were employed for regression tasks. The resulting AI (artificial intelligence)-driven hybrid models enable rapid prediction and optimization of EMS design and operating parameters, reducing computational and experimental costs. This research is situated at the intersection of membrane science, artificial intelligence, and computational modeling, forming part of a broader foresight agenda aimed at developing next-generation intelligent membranes and adaptive control strategies for sustainable water treatment. The methodology provides a scalable framework for integrating physically based modeling and machine learning into the design of high-performance electromembrane systems.

## 1. Introduction

The modern era is characterized by the rapid development of artificial intelligence (AI) technologies, which are actively penetrating many areas of human activity, including membrane electrochemistry [1,2,3,4,5,6,7,8,9,10]. For example, Abuwatfa, AlSawaftah et al. [1] present a systematic review of the application of artificial neural networks to predict membrane fouling in pressure-driven processes (microfiltration, ultrafiltration, nanofiltration, and reverse osmosis). The work demonstrates that artificial neural networks (ANNs), thanks to their nonlinear mapping capabilities, can effectively capture complex relationships between process parameters and fouling development, outperforming traditional empirical and mechanistic models. The article discusses the advantages and limitations of ANN approaches, identifying the potential of the method for a deeper understanding of fouling mechanisms.

The authors of [2] present an updated Python package−PiNN 1.0, with the equivariant neural network PiNet2, designed for atomistic modeling of electrochemical systems. PiNet2 integrates vector (P3) and tensor (P5) equivariant features into graph convolutional networks, significantly improving the prediction accuracy of energy, forces, dipoles, and polarizability. Thus, PiNN is positioned as a high-performance platform for electrochemistry, combining ML potentials with electrical boundary conditions, outperforming DeePMD and MACE in specialized tasks.

Di Martino, Abraham, Pistikopoulos, and colleagues [3] applied rectified linear unit (ReLU) artificial neural networks to comprehensively optimize the performance of industrial reverse osmosis plants based on a superstructure approach. ANNs are used as surrogate models to quickly calculate the performance of various plant configurations, enabling engineers to perform global optimization while minimizing energy and capital costs. The model achieved exceptionally high accuracy in predicting plant current, salt drift, and other critical parameters, demonstrating the practical value of the approach for desalination plant design and operation.

The paper [4] analyzes modern applications of artificial intelligence in wastewater treatment, including membrane technologies. The paper describes in detail the key tasks solved using AI: pollutant monitoring, prediction of treatment efficiency, membrane biofouling management, and energy consumption optimization. The authors analyze current trends: the integration of AI with sensor systems, the use of machine learning to analyze large volumes of data, and the automation of decision-making at treatment plants. The paper also examines the prospects for using AI to improve the sustainability and environmental friendliness of processes, as well as for the development of new combined technologies, such as cascade treatment systems and closed water management cycles.

The authors of [5] review current methods for predicting fouling in membrane bioreactors (MBRs), where membrane fouling remains a major barrier to the widespread use of this highly effective wastewater treatment technology. The paper critically analyzes the use of artificial neural networks and hybrid approaches for fouling prediction, discussing key challenges—including data quality, the risk of overfitting, and model interpretability issues. The article provides practical recommendations for the successful application of ANNs, emphasizing the need for combining multiple methods, long-term data collection, and continuous model improvement.

In [6], the authors present AI algorithms and a corresponding computing platform designed to predict membrane fouling processes in anaerobic membrane bioreactors (AnMBRs) used for wastewater treatment. The input factors included operating parameters, biomass properties, and membrane characteristics. The use of ANN and random forest methods made it possible to confirm the influence of individual factors and their interactions on the membrane fouling process, identifying important correlations necessary for optimizing their operation. The work [7] is devoted to the development of effective forecasting tools facilitating the implementation of organic solvent nanofiltration technology. An extensive dataset was compiled, including more than 18 characteristics, based on which AI models (neural networks, support vector machines, and random forest) were built to estimate the retention and transmission of molecules. Principal component analysis (PCA) showed the similarity of key factors determining both of these characteristics. The study [8] describes a comprehensive mathematical model for improving the performance of reverse osmosis systems in treating brackish water with moderate to high salt content. This model is based on the RSM and ANN approach, developed specifically for improving the performance of RO systems. The use of ANN showed significant superiority over the RSM model in terms of prediction accuracy. A two-stage optimization procedure made it possible to improve the system performance by achieving an optimal balance between the performance index and the energy consumption. The paper [9] discusses the use of a three-layer residual artificial neural network (R-TNN) for predicting the performance of thin-film nanocomposite reverse osmosis membranes (TFN-ROM). The resulting model demonstrated the ability to accurately account for the complex relationship between membrane permeability and retention capacity, which allows for the creation of highly efficient membrane structures. Finally, work [10] presents a deep learning protocol for predicting the electrical conductivity of hydroxide ions (OH-) in anion-exchange membranes (AEMs) based on poly (2,6-dimethylphenylene oxide) with functionalized ionic groups. The developed model achieves an accuracy of approximately 99.7%, which opens new prospects for the design of membranes with predetermined properties. This study emphasizes the effectiveness of an approach that consists of replacing standard physical variables with complex quantities constructed from these same quantities in specific combinations determined by the nature of the phenomena under study. This approach has several important advantages. First, a significant reduction in the number of independent variables facilitates the formulation and solution of the problem. Second, the use of complex variables that simultaneously integrate multiple influences of factors allows for a more detailed understanding of the internal structure and interrelations characteristic of the processes under study. As a result, the general form of the equations and graphical representation becomes more transparent and informative.

Another remarkable feature of complex variables is the fact that each fixed value of such a variable can correspond to an infinite number of different combinations of the basic quantities. Thus, each point in the space of generalized variables actually reflects an entire family of equivalent states united by common qualitative features. This fact allows for the simultaneous analysis of not just isolated particular cases, but entire classes of similar processes, increasing the level of generality of the conclusions obtained. The use of generalized dimensionless variables forms the basis of a specialized scientific field called similarity theory and dimensional analysis [11]. Although this approach has long been successfully used in physics, its adaptation to chemical disciplines remains an underdeveloped area of science. The goal of this work is to demonstrate the potential for applying similarity theory directly to membrane electrochemistry, revealing a fundamentally new perspective on traditional problems and opening broad horizons for a deeper understanding of the mechanisms of ongoing processes. As is well known, water is a vital resource for life, and the primary solution to the problem of freshwater shortage is access to effective desalination technologies, which include, in particular, electromembrane technologies. The efficiency of electromembrane systems for water purification, such as electrodialysis, is highly dependent on the hydrodynamics of the process, as well as the properties of the membrane surface. To reduce the costs of EMS experiments to intensify desalination, it is necessary to develop mathematical models of EMS water purification. Electroconvection is the primary mechanism of over-limit transport in membrane systems, and therefore, its intensification should be considered: an earlier onset to reduce the “plateau,” an increase in the size of electroconvective vortices, etc. When analyzing electroconvection processes, one must deal with a large number of diverse variables, such as the initial concentration of the substance, the change in electrical potential, the solution feed rate, the channel width, and other parameters, each of which acts as a separate factor. However, the influence of the specified parameters is not manifested individually, but in a complex, forming certain dimensionless quantities (criterion numbers, trivial similarity criteria such as Reynolds, Peclet numbers, etc.), and the number of parameters decreases, according to the theorem of similarity theory. Criterion numbers characterize internal connections, for example, the Reynolds number characterizes the ratio of inertial forces to friction forces, and the Peclet number the ratio of convective transfer to diffusion, etc., and, depending on their value, determine the qualitative and quantitative picture of the solution flow and the transfer process. Moreover, the relationship between the physical parameters of the electroconvection process can be represented as a relationship between the similarity criteria, including such characteristics important for intensification as, for example, the dimensionless diameter of the electroconvective vortex (ECV), the length of the plateau of the current–voltage characteristic, *ECV_CEM_*—the beginning of electroconvection near the CEM, *ECV_AEM_*—the beginning of electroconvection near AEM.

(1) in the potentiostatic case we have (using the example of the dimensionless diameter of an electroconvective vortex *D_ecv_*):
  Decv=f(Re,Pe,ε,Kel,dφ)
  where $d_{\varphi }$—dimensionless potential jump;
(2) in the potentiodynamic case we have:
  $$D_{ecv}=f(Re,Pe,\varepsilon ,K_{el},d_{1})$$
  where $d_{1}$—dimensionless potential sweep rate. Besides, *Re*—Reynolds number, *Pe*—Peclet number, ε—this is the ratio of the square of the thickness of the equilibrium space charge region to the square of the distance between the membranes [12,13], and $K_{el}$—this is the ratio of the electrical force to the inertial force. Typically, to construct functions like f either an exact solution to the boundary value problem of a mathematical model or experimental studies are used. Currently, finding an analytical solution to the electroconvection problem is difficult due to insurmountable mathematical difficulties, and an experimental determination is extremely labor-intensive due to the large number of criterion numbers. In this regard, this paper proposes using neural networks to determine functional dependencies. To implement neural networks, a sample was compiled based on numerical experiments with the developed mathematical model. The entire sample was divided into two sets: a set used for training (setting weights and biases) and a test set, which was used to determine the correctness of the neural network. A multilayer feedforward network was chosen as the network architecture; the backpropagation algorithm with different optimizers was used to train the neural network. The application of the functions found will be used in the future to calculate the optimal geometric and technological parameters of the desalination process in electromembrane units.

## 2. Methods

### 2.1. Mathematical Model

**Figure 1:**
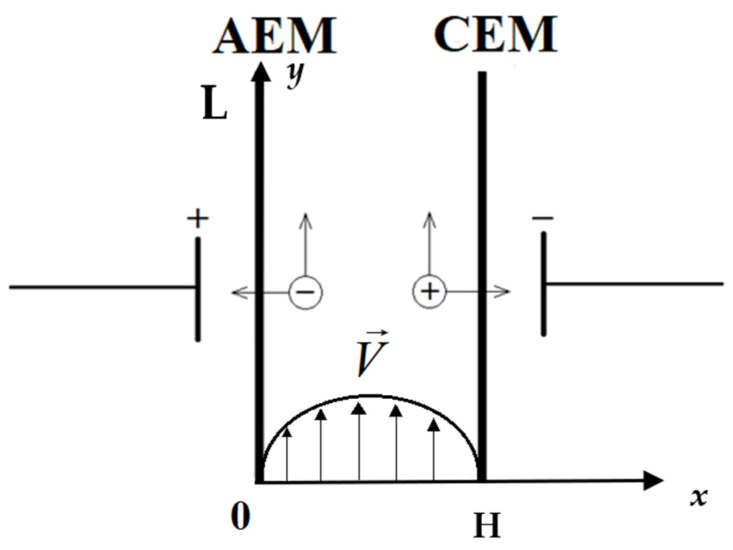
Two-dimensional diagram of the representation of the desalination channel (DC) in an electrical circuit. Designations: AEM—anion exchange membrane, CEM—cation exchange membrane; arrows indicate possible directions of anion migration. (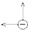) and cation (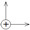), H—channel width, L—channel length. The Poiseuille parabola is shown below, which is the initial distribution of the solution flow velocity along the desalination channel; the solution flow is then calculated using a mathematical model.

The flow of liquid in a rectangular channel of length L and width H, equipped with cation-exchange and anion-exchange membranes, is studied; a schematic representation is shown in Figure 1. Modeling the dynamics of liquid flow in a flow channel, taking into account the effects of electroconvection, is carried out by means of a joint solution of the system of Nernst–Planck–Poisson equations (Formulas (1)–(4)) and the Navier–Stokes equations (Formulas (5) and (6)).

(1) $${\overset{\to }{j}}_{i}=-\frac{F}{RT}z_{i}D_{i}C_{i}\overset{\to }{E}-D_{i}\nabla C_{i}+C_{i}\overset{\to }{V},\;i=1,2$$

(2) $$\frac{\partial C_{i}}{\partial t}=-div{\overset{\to }{j}}_{i},\;i=1,2$$

(3) $$\varepsilon_{r}\Delta \varphi =-F(z_{1}C_{1}+z_{2}C_{2})$$

(4) $$\overset{\to }{I}=F(z_{1}{\overset{\to }{j}}_{1}+z_{2}{\overset{\to }{j}}_{2})$$

(5) $$\frac{\partial \overset{\to }{V}}{\partial t}+(\overset{\to }{V}\nabla )\overset{\to }{V}=-\frac{1}{\rho_{0}}\nabla P+\nu \Delta \overset{\to }{V}+\frac{1}{\rho_{0}}\overset{\to }{f}$$

(6) $$div(\overset{\to }{V})=0,$$

where

$$\overset{\to }{f}=\rho \overset{\to }{E}=-\varepsilon_{r}\Delta \varphi \overset{\to }{E}=\varepsilon_{r}\Delta \varphi \nabla \varphi =\varepsilon_{r}\overset{\to }{E}div\overset{\to }{E}.$$

Here, $\overset{\to }{V}$—flow velocity of the solution; Index 1 refers to cations, index 2 refers to anions; ${\overset{\to }{j}}_{1},{\overset{\to }{j}}_{2}$—fluxes; $C_{1},C_{2}$—concentrations in the solution; $z_{1},z_{2}$—charge numbers; $\overset{\to }{I}$—current density; $D_{1},D_{2}$—diffusion coefficients; φ—electric potential field; $\overset{\to }{E}=-\nabla \varphi$—electric field strength; $\varepsilon_{r}$—dielectric permittivity of the electrolyte; F—Faraday’s constant; R—universal gas constant; T—absolute temperature; t—time; $\rho_{0}$—density; ν—kinematic viscosity; P—pressure; $\rho =F(z_{1}C_{1}+z_{2}C_{2})$—distribution of charge densities.

The potentiodynamic regime is being investigated. The boundary conditions are set within the following constraints:

(1) Boundary conditions on the membrane surfaces
  $$C_{2}\left(t,0,y\right)=C_{2m},\;\left(\frac{\partial C_{1}}{\partial x}-\frac{F}{RT}C_{1}E\right)(t,0,y)=0,\;\varphi \left(t,0,y\right)=0$$
  $$C_{1}\left(t,H,y\right)=C_{1m}(t),\;\left(\frac{\partial C_{2}}{\partial x}+\frac{F}{RT}C_{2}E\right)(t,H,y)=0,\;\varphi (t,H,y)=\Delta \varphi (t)$$
  $$C_{1}\left(0,x,y\right)=C_{0},\;C_{2}\left(0,x,y\right)=C_{0},\;\varphi \left(0,x,y\right)=0,$$
  where Δφ(t) denotes a potential jump similar to that indicated earlier and is traditionally performed $\Delta \varphi (t)=-d_{1}\cdot t$. The rate of increase in the potential jump is determined by the parameter d_1_, whose unit is volts per second [V/s].

The no-slip condition is assumed for the fluid velocity on the channel walls.

(2) Boundary conditions at the channel inlet: At the inlet to the desalination channel, specified ion concentrations are established that satisfy the condition of electroneutrality, that is:
  $$C_{i}(t,x,0)=C_{i,0}\;\;i=1,2$$
  (7) φ(t,x,0)=Δφ⋅x/H

A constant concentration distribution, coupled with Ohm’s law, leads to the expression presented in Formula (7). Consequently, this formula is consistent with both Ohm’s law and the condition of constant concentrations at the channel entrance.

Distribution of solution flow rate $\overset{\to }{V}$ at the input we accept the corresponding Poiseuille law, according to which the velocity profile has a parabolic form, but $V_{0}$ denotes the average flow rate of the solution.

(8) $$V_{x}=0,\;V_{y}=6V_{0}\frac{x}{H}(1-\frac{x}{H}).$$

(3) Boundary conditions at the outlet of the desalination channel (DC):
  (a) At the boundary of the output region y=L,x∈[0,H],t≥0 for component concentrations, a condition ensuring conservation of ion flux is imposed, meaning that removal of salt ions from the DC area happens solely due to convective transport:
    $$-\overset{\to }{n}\cdot {\overset{\to }{j}}_{i}(x,L,t)=-V_{y}(x,L,t)C_{i}(x,L,t),i=1,2$$
  (b) For the potential jump the following condition is set:
    $$-\overset{\to }{n}\cdot \nabla \varphi =0,$$
    i.e.,
    $$\frac{\partial \varphi (x,L,t)}{\partial y}=0$$

(4) The initial conditions are set as follows: For concentrations and electric potential:

$$C_{i}(0,x,y)=C_{i,0}\;i=1,2.$$

φ(0,x,y)=Δφ⋅x/H

We assume that the flow velocity of the liquid is distributed according to Poiseuille’s law (8).

### 2.2. Similarity Method for Transport Processes in a Flat Channel

This work continues and develops the ideas presented in studies [14,15,16], where a theory of process similarity was developed for a two-dimensional desalination channel.

1. Conversion to dimensionless form

We select characteristic parameters as the basic scaling quantities $C_{0},V_{0},d_{\varphi },H$ and we will make the transition to a dimensionless representation, considering the following expressions to be valid:

$$x^{\left(u\right)}=\frac{x}{H};\;y^{\left(u\right)}=\frac{y}{H};\;L^{\left(u\right)}=\frac{L}{H};t^{\left(u\right)}=\frac{tV_{0}}{H};\;{\overset{\to }{V}}^{\left(u\right)}=\frac{\overset{\to }{V}}{V_{0}};\;P^{\left(u\right)}=\frac{P}{\rho_{0}V_{0}};$$

$${\overset{\to }{E}}^{\left(u\right)}=\frac{HF}{RT_{0}}\overset{\to }{E};\;D_{i}^{\left(u\right)}=\frac{D_{i}}{D_{0}},\;{i_{av}}^{\left(u\right)}=\frac{i_{av}H}{DC_{0}F};\;\eta^{\left(u\right)}=\frac{\eta }{DC_{0}F};\;f_{0}=\frac{\rho_{0}V_{0}^{2}}{H};$$

$$d_{\phi }^{\left(u\right)}=\frac{F}{RT_{0}}d_{\phi };\;\varphi^{\left(u\right)}=\frac{F}{RT_{0}}\varphi ;\varepsilon^{\left(u\right)}=\frac{RT_{0}\varepsilon }{F^{2}H^{2}C_{0}};\;\;C_{i}^{\left(u\right)}=\frac{C_{i}}{C_{0}};$$

$${\overset{\to }{j}}_{i}^{\left(u\right)}=\frac{H}{D_{0}C_{0}}{\overset{\to }{j}}_{i};\;{\overset{\to }{I}}^{\left(u\right)}=\frac{H}{D_{0}C_{0}F}\overset{\to }{I};$$

where $D_{0}=D_{1}$—average diffusion coefficient, and the superscript (*u*) denotes that the quantity under consideration is dimensionless.

2. Physical meaning of dimensionless parameters in equations and boundary conditions

In paper [15], the boundary value problem associated with model [14] was reformulated into a dimensionless form by utilizing the characteristic scaling factors mentioned earlier. This transformation led to the introduction of several key dimensionless parameters:

$L^{\left(u\right)}=\frac{L}{H}$—representing the dimensionless channel length, $Pe=\frac{V_{0}H}{D_{0}}$—known as the Peclet number, indicating the ratio of convective transport intensity to molecular diffusion, Re=V0Hν—referred to as the Reynolds number, illustrating the equilibrium between inertial $F_{in}=\rho_{0}H^{2}V_{0}^{2}$ and viscous friction forces $F_{tr}=\nu \rho_{0}V_{0}H$, along with additional dimensionless numbers $\varepsilon^{\left(u\right)}=\frac{RT\varepsilon }{H^{2}C_{0}F^{2}}$ and $K_{el}=\frac{RTC_{0}}{\rho_{0}V_{0}^{2}}$.

3. Physical interpretation of the quantity $\varepsilon^{\left(u\right)}$

This parameter, first introduced in [12,13], has received the following physical interpretation. The formula can be represented as follows: $\varepsilon^{\left(u\right)}=\frac{RT\varepsilon }{H^{2}C_{0}F^{2}}=2{\left[\frac{l_{d}}{H}\right]}^{2}$, where $l_{d}=\sqrt{\frac{RT\varepsilon }{2C_{0}F^{2}}}$—Debye length, which characterizes the size of the equilibrium space charge region.

Thus, the parameter $\varepsilon^{\left(u\right)}$ is the ratio of the square of twice the Debye length (the thickness of the space charge region) to the square of the intermembrane distance, or equivalently, the ratio of the Debye length itself to half the intermembrane distance. Physically, this means that the parameter $\varepsilon^{\left(u\right)}$ determines the ratio of the sizes of the charged and uncharged zones of the solution within the intermembrane gap.

4. Physical interpretation of the quantity $K_{el}=\frac{RTC_{0}}{\rho_{0}V_{0}^{2}}$

When the fluid flows with an average velocity $V_{0}$, the quantity $P_{0}=\rho_{0}V_{0}^{2}$ serves as a characteristic pressure, often referred to as the velocity head or dynamic pressure. It is frequently utilized in both hydrodynamics and aerodynamics as a scaling factor for hydrodynamic pressures and appears in various aerodynamic coefficients.

The electric pressure acting on a unit cross-sectional area is written as:

$$P_{el}=2RTC_{0}$$

In addition, the value $RTC_{0}$, according to Van’t Hoff’s law corresponds to the osmotic pressure of a solution with a concentration of $C_{0}$.

Based on this, the value of the ratio $K_{el}=\frac{P_{el}}{P_{0}}$ can be interpreted as the ratio of the characteristic pressure caused by the action of electrical force to the characteristic hydrodynamic pressure arising as a result of the movement of the solution.

5. $K_{ek}$—the criterion number of electroconvection. It is defined as the coefficient of the dimensionless electric force driving the generation of electroconvective flows. Its expression in terms of physical parameters is as follows:

$$K_{ek}=\varepsilon^{\left(u\right)}K_{el}.$$

Then, expressing both components through dimensional parameters, we obtain:

$$K_{ek}=2{\left[\frac{l_{d}}{H}\right]}^{2}\frac{RTC_{0}}{\rho_{0}V_{0}^{2}}=2\frac{RTC_{0}l_{d}^{2}}{\rho_{0}V_{0}^{2}H^{2}}=2\frac{F_{el}}{F_{in}},$$

where $F_{el}$—electric force acting on a space charge region, and $F_{in}$—force of inertia.

This expression shows that the criterion number of electroconvection $K_{ek}$ is the ratio of electrical force to inertial force.

It is important to note that the inertial force does not depend on the initial concentration *C*_0_, since it is determined by the kinetic properties of the liquid. On the other hand, the electric force $F_{el}$, being the product of the electric pressure and the space charge volume, also turns out to be independent of the initial concentration. Increasing the concentration increases the electric pressure but simultaneously narrows the space charge volume, which mutually compensates for the effect of the initial concentration.

$$F_{el}=RTC_{0}l_{d}^{2}=RTC_{0}\frac{RT\varepsilon }{2C_{0}F^{2}}=\frac{{\left(RT\right)}^{2}\varepsilon }{2F^{2}}$$

Therefore, the criterion number of electroconvection $K_{ek}$ does not actually depend explicitly on the initial concentration, which confirms the assumption about the weak connection between electroconvection and concentration.

The definition of the number $K_{ek}$ implies the presence of a certain critical value ${\overset{¯}{K}}_{ek}$, upon exceeding which the widespread formation of electroconvective structures in the channel begins.

An assessment of the dimensionless parameters reveals that for typical values of dimensional quantities relevant to electrodialysis, the Peclet and Reynolds numbers fall within the orders of magnitude of approximately $10^{2}÷10^{4}$ and 1÷100, respectively. Additionally, the parameter ε spans a range from $10^{-17}$ to $10^{-7}$, making it possible to treat it as a small parameter.

The number $K_{el}$ is of the order of $10÷10^{3}$, and the number $K_{ek}$ is of the order of $10^{-14}÷10^{-4}$.

6. In addition to the dimensionless parameters considered earlier, additional dimensionless quantities are introduced, appearing in the boundary conditions [15]:

$d_{\varphi }^{\left(u\right)}=\frac{F}{RT_{0}}d_{\varphi }$—total potential value;

$d_{0}^{\left(u\right)}=\frac{F}{RT_{0}}d_{0}$—initial value of potential;

$d_{1}^{\left(u\right)}=\frac{F}{RT_{0}}d_{1}\cdot t$—growth rate of potential;

$C_{km}^{\left(u\right)}=\frac{C_{km}}{C_{0}}$—limit value of concentration on a CEM;

$C_{am}^{\left(u\right)}=\frac{C_{am}}{C_{0}}$—limit value of concentration on the AEM.

These parameters reflect the physical conditions present at the boundaries of the channel and guarantee proper formulation of the boundary value problem. It is postulated that the initial concentration within the channel matches the concentration at the channel inlet. For the sake of simplification in subsequent computational experiments, a unified initial concentration equivalent to the entry concentration has been implemented. $C_{am}=C_{km}=C_{0}$.

7. Dimensionless equations and boundary conditions

Upon transitioning to dimensionless variables and parameters, the system of Nernst—Planck—Poisson and Navier—Stokes equations, originally stated in [14], takes the following form (for notational convenience, the dimensionless index “u” is dropped throughout):

$${\overset{\to }{J}}_{i}=z_{i}D_{i}C_{i}\overset{\to }{E}-D_{i}\nabla \;C_{i}+PeC_{i}\overset{\to }{V},\;\;i=1,2$$

$$Pe\frac{\partial \;C_{i}}{\partial t}=-div{\overset{⇀}{j}}_{i},\;i=1,2$$

$$\varepsilon \Delta \varphi =-\left(z_{1}C_{1}+z_{2}C_{2}\right)$$

$$\overset{\to }{I}=z_{1}{\overset{\to }{j}}_{1}+z_{2}{\overset{\to }{j}}_{2}$$

∂V→∂t+(V→∇)V→=−∇P+1ReΔV→+εKelΔφ∇φ

$$div(\overset{\to }{V})=0$$

The Nernst–Planck–Poisson and Navier–Stokes equations embody expressions of basic conservation principles. Consequently, the comprehensive model describing ion transport incorporating electroconvection, beyond merely defining particular equations, primarily hinges on the specification of appropriate boundary conditions. Detailed information regarding dimensional boundary conditions is available in [14]. These include:

x=0—the boundary between the surface of the AEM and the solution;

x=1—the boundary between the surface of the CEM and the solution;

y=0—inlet to the desalination channel;

y=L—outlet from the desalination channel.

Let us now write the dimensionless boundary conditions considering the previously introduced dimensionless parameters.

1. Conditions on Ion Exchange Membrane Surfaces

(a) Surface of Anion Exchange Membrane: (x=0,y∈[0,L],t≥0):

$$C_{2}(t,0,y)=C_{am}$$

Additionally, we presume that the AEM exhibits ideal selectivity and equipotentiality characteristics:

$$\left(\frac{\partial C_{1}}{\partial x}+z_{1}C_{1}\frac{\partial \varphi }{\partial x}\right)(t,0,y)=0.$$

φ(t,0,y)=0

(b) Surface of Cation Exchange Membrane: (x=1,y∈[0,L],t≥0):

$$C_{1}(t,1,y)=C_{km}$$

$$\left(\frac{\partial C_{2}}{\partial x}+z_{2}C_{2}\frac{\partial \varphi }{\partial x}\right)(t,1,y)=0.$$

$$\varphi (t,1,y)=d_{\varphi }(t),$$

where $d_{\varphi }=d_{0}+d_{1}\cdot t$, t≥0,y∈[0,L], $d_{0}$—the initial value of the potential jump and $d_{1}$—the growth rate of the potential jump.

A no-slip condition is imposed on the membrane surface, meaning zero velocity of the liquid relative to the walls:

$$\begin{array}{l} V_{x}(t,0,y)=0,\;V_{y}(t,0,y)=0,\\ V_{x}(t,1,y)=0,\;V_{y}(t,1,y)=0 \end{array}$$

2. At the inlet to the DC $x\in \left[0,1\right],\;y=0,\;t\geq 0$

At the entrance to the channel, the concentrations of sodium and chlorine ions are assumed to be uniformly distributed, and the condition of electrical neutrality is assumed to be met, that is, the equality of positive and negative charges.

$$C_{i}(t,x,0)=1,\;i=1,2.$$

The condition is imposed on the potential:

$$\frac{\partial \varphi (t,x,0)}{\partial y}=-\frac{1}{z_{1}^{2}D_{1}+z_{2}^{2}D_{2}}\left(z_{1}D_{1}\frac{\partial C_{1}(t,x,0)}{\partial y}\right.\left.+z_{2}D_{2}\frac{\partial C_{2}(t,x,0)}{\partial y}\right)$$

ensuring the absence of currents.

The velocity profile at the channel entrance has a parabolic shape corresponding to the Poiseuille distribution.

$$V_{x}\left(t,x,0\right)=0,\;V_{y}\left(t,x,0\right)=6x\left(1-x\right)$$

3. At the outlet of the DC $x\in \left[0,1\right],\;y=L,\;t\geq 0$

For concentrations, we’ll apply the condition for ion flow at the outlet, assuming that the removal of salt ions from the DC results purely from the flow of the solution itself:

$$z_{i}C_{i}(t,x,L)\frac{\partial \varphi (t,x,L)}{\partial y}+\frac{\partial C_{i}(t,x,L)}{\partial y}=0\;\;\;i=1,2$$

$$\frac{\partial \varphi (t,x,L)}{\partial y}=0$$

Velocity conditions at the inlet and outlet are alike:

$$V_{x}(x,L,t)=0,V_{y}(x,L,t)=6x\left(1-x\right).$$

Moreover, at the corners of the channel’s outlet, an extra pressure condition is applied, permitting free departure of vortex formations from the desalination channel domain.

4. Initial Conditions

Initial conditions (t=0) conform to the given boundary conditions. As an illustration, the initial ion concentration inside the channel aligns with the concentration level at the channel inlet, and for the potential φ the initial condition can be a simple linear function independent of y:

$$\varphi (0,x,y)=d_{\varphi }x$$

Such conditions ensure a smooth start of the modeling process and contribute to rapid stabilization of the numerical solution.

The boundary value problem was solved using the finite element method implemented in Comsol Multiphysics 6.2 with an adaptive time step. PARDISO, a high-performance direct solver for sparse linear systems of equations, was chosen as the solver. It is designed for the efficient solution of large problems in scientific and engineering calculations. It supports both serial and parallel modes of operation, making it particularly useful for problems with high computational complexity [17,18].

According to the above reasoning, electroconvection depends not explicitly, but indirectly on the initial concentration $C_{0}$ (and boundary concentrations $C_{am}$, $C_{km}$).

**Figure 2:**
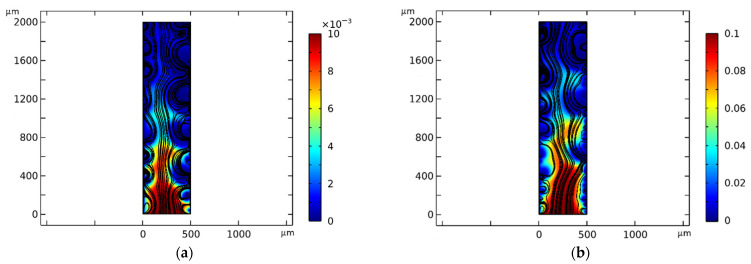
Flow at the same potential drop and different concentrations $C_{am}=C_{km}=C_{0}$ at: (**a**) $C_{0}=0.01$ mol/m^3^; (**b**) $C_{0}=0.1$ mol/m^3^. The calculations are shown for a time of t = 140 s, which is equivalent to a potential jump of 1.75 V.

It has been experimentally established (Figure 2) that increasing the boundary concentrations $C_{am}=C_{km}=C_{0}$ by a factor of 10 results in only a slight change in the size of electroconvective vortices, measured along the outer contour of the ion trajectory envelope. This is due to the fact that the main factors determining the intensity of electroconvection are the hydrodynamic characteristics of the flow and the electric field, and not the concentration of the salt solution itself.

**Figure 3:**
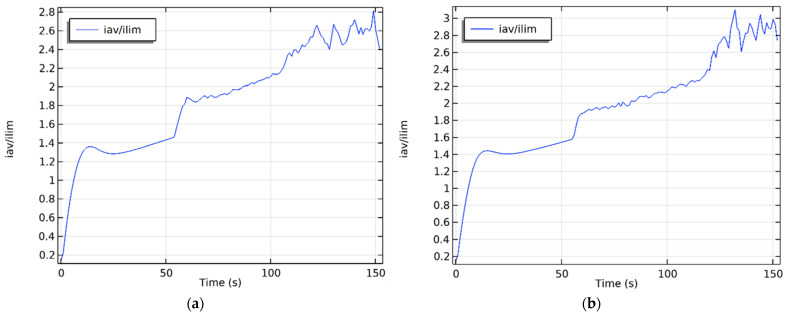
Current–voltage characteristics at a potential sweep rate of 0.0125 V/s (dimensionless parameter d_1_ = 0.49) and different values of the initial and boundary concentrations at different values of initial and boundary concentrations $C_{am}=C_{km}=C_{0}$ at: (**a**) $C_{0}=0.01$ mol/m^3^; (**b**) $C_{0}=0.1$ mol/m^3^. The ordinate axis shows the ratio of current to maximum current.

The influence of boundary concentrations $C_{am}=C_{km}=C_{0}$ on the current–voltage characteristic is also insignificant (Figure 3).

The protrusion (local maximum) between the ohmic region and the plateau in Figure 3a,b is explained by the non-stationarity effects caused by the high sweep speed *d*_1_ = 0.49 (see Section 3.1).

## 3. Results

### 3.1. Training Set for Constructing a Neural Network

Important characteristics of the electroconvective transfer process include the dimensionless diameter of the ECV, the length of the CVC plateau, the onset of electroconvection near the AEM, and the onset of electroconvection near the CEM. These characteristics are determined through a computational experiment, followed by recording of the corresponding values. However, conducting such computational experiments is time-consuming and resource-intensive. For example, conducting similar experiments in this study took from several days to several weeks, depending on the input parameters. Therefore, for such problems, it is advisable to learn how to predict the required characteristics while avoiding direct calculations. Neural networks can be used for this purpose [19], after first collecting a representative training sample through direct calculations.

Since the initial concentration has little effect on the sizes of the ECV and CVC, when conducting computational experiments to collect information for the training sample, it was decided to vary the potential sweep rate and the solution pumping rate, and the corresponding criterion numbers will also change.

Thus, the task is to find the following functional dependencies:

Decv=f(Re,Pe,Kel,d1)—dimensionless diameter of an electroconvective vortex;

Lpl=p(Re,Pe,Kel,d1)—length of the plateau of the current voltage characteristic;

ECVAEM=h(Re,Pe,Kel,d1)—the beginning of electroconvection near the AEM;

ECVCEM=g(Re,Pe,Kel,d1)—the beginning of electroconvection near the CEM, here $d_{1}$—dimensionless potential sweep rate.

**Figure 4:**
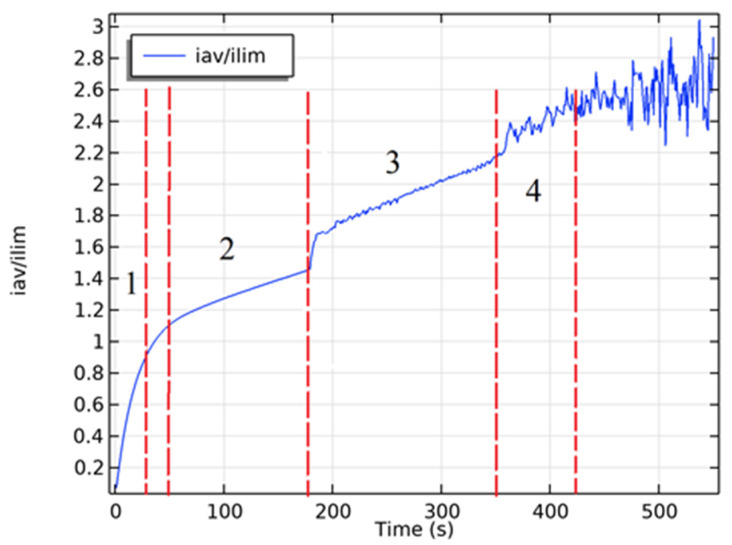
An example of a current–voltage characteristic divided into several regions: 1—initial linear region; 2—plateau of the maximum current; 3—occurrence of unstable ECV only on CEM; 4—occurrence of unstable ECV simultaneously on CEM and AEM. The red dashed lines in the figure indicate the boundaries of the regions.

Let us first vary the potential sweep rate, fix the initial concentration in $C_{0}=0.01$ mol/m^3^ and the initial velocity in $V_{0}=10^{-4}$ m/s. We will determine the ECV diameter, the CVC plateau length, and the onset of electroconvection near the AEM and CEM, first in dimensional form, and then nondimensionalize using the formulas given in Section 2.2 of this study. To determine the plateau length, it is necessary to plot the CVC characteristic. Figure 4 shows the current–voltage characteristic at a potential sweep rate of 0.00375 V/s. *L_pl_* was determined from region 2 of the CVC characteristic, and the *ECV_CEM_* and *ECV_AEM_* parameters from regions 3 and 4, respectively.

**Figure 5:**
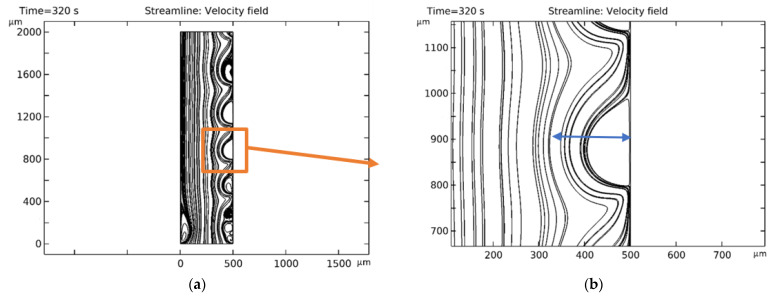
Determining the region of the ECV by the solution flow lines: (**a**) selecting the region in the center of the CEM; (**b**) enlargement of (**a**)—determining the diameter of the ECV in the selected area by the outer envelope of the flow lines (the scale is not respected).

The onset of electroconvection on the membranes was also confirmed by the graph of the solution flow lines, and the diameter of the ECV was also determined from it (Figure 5).

As a result of conducting a series of computational experiments with different potential sweep rates and calculating the required characteristics in dimensionless form, we obtained Table 1.

**Figure 6:**
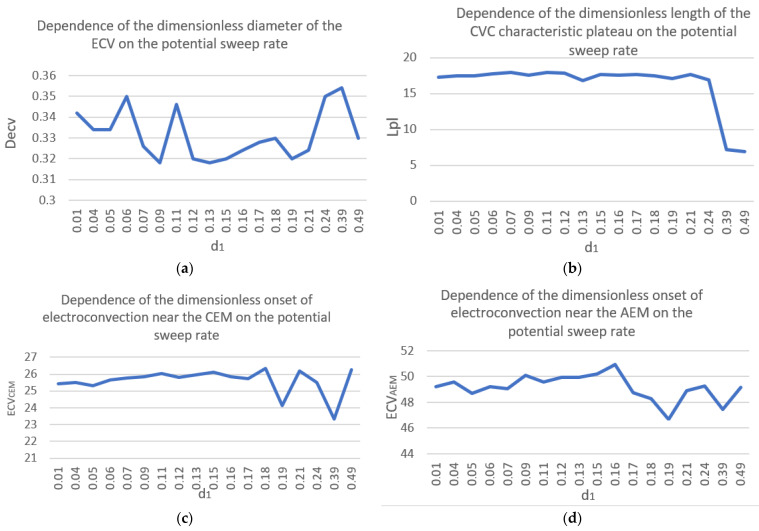
Dependence of the sought characteristics on the dimensionless potential sweep rate: (**a**) *D_ecv_*; (**b**) *L_pl_*; (**c**) *ECV_CEM_*; (**d**) *ECV_AEM_*.

Let us plot graphs of the dependence of the found parameters on the dimensionless potential sweep rate (Figure 6). Figure 6a suggests that the dimensionless diameter of the ECV is virtually independent of the potential sweep rate and varies from 0.32 to 0.36. The length of the CVC plateau is virtually constant at low sweep rates, but then decreases significantly at high d_1_ (Figure 6b). The parameters of the onset of electroconvection on the CEM and AEM behave stably at low potential sweep rates, but at high values, their character becomes abrupt (Figure 6c,d). Thus, it can be concluded that a quasi-stationary regime is realized at low sweep rates (0.01 to 0.07), beyond which the desalination process becomes non-stationary. One manifestation of these nonstationarity effects, as seen by comparing the CVC in Figure 3 and Figure 4, is the appearance of a small hump near the limiting current on the CVC.

Table 1 was then expanded to Table 2 by conducting a series of experiments with the initial velocity and potential sweep rate at a constant initial concentration of $C_{0}=0.01$ mol/m^3^. The data from this table served as the training set for the neural network being developed.

### 3.2. Building a Neural Network

The neural network being developed should solve the task of simultaneously predicting four parameters (multi-output regression); however, before feeding the training sample (Table 2) into the neural network, preprocessing of the data must be performed (removal of duplicate values and outliers, splitting the sample into training and test, normalization).

Data normalization plays an important role in the training process of neural networks. It helps significantly increase training efficiency and improve the quality of the resulting models. Normalization is necessary for:

1. Optimizing training speed

Neural networks use the gradient descent method to minimize the objective function. Gradients are calculated relative to changes in weights, and if the input data have different scales, this slows down the training process. The greater the difference in feature scales, the longer it will take for the model to converge.

2. Improving accuracy and robustness

Most algorithms depend on the distance between features, and differences in scale can distort the distance between elements, negatively impacting model quality.

3. Addressing multicollinearity

Some features can be correlated with each other, degrading model performance.

**Figure 7:**
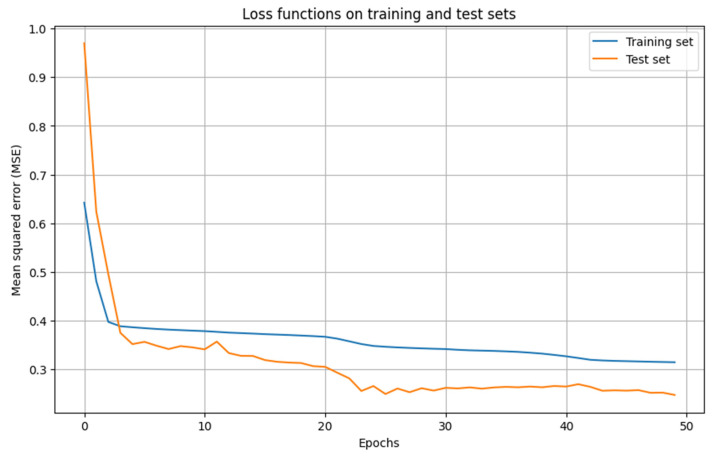
Loss function graphs for training and test sets.

**Figure 8:**
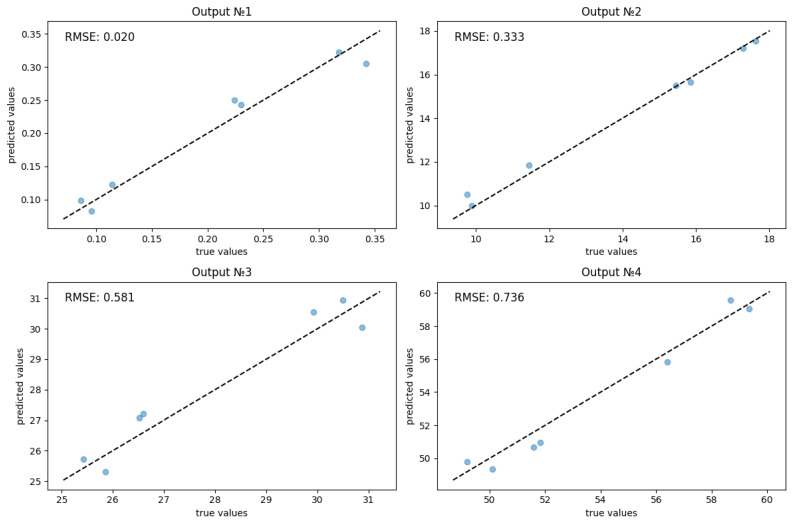
Predicted and actual values for output variables: D_ecv_ (Output No.1), L_pl_ (Output No.2), ECV_CEM_ (Output No.3), ECV_AEM_ (Output No.4). The dashed lines indicates the ideal regression line.

Taking into account the above reasons, a standard normalization (standardization) of the training sample was performed. After this, a data split ratio was chosen: 80% for training, 20% for testing, and cross-validation was performed into five parts. Cross-validation is necessary due to the small data volume, the need for an accurate final estimate, and when comparing multiple neural network parameter configurations for objective comparison. When selecting neural network parameters such as the number of layers, neurons, learning rate, activation functions, and optimizers, it is useful to use hyperparameter selection using Grid Search or Randomized Search. Since searching for the best hyperparameters by randomly selecting them from a given distribution of possible values is more efficient than Grid Search for large hyperparameter spaces, Randomized Search was chosen. The most popular loss function for regression problems, MSE (Mean Square Error) [20,21], was used as the loss function, and training lasted 50 epochs. As a result of hyperparameter optimization, the best performance was achieved using an architecture of 4-3-4: the input layer has 4 neurons, one hidden layer with 3 neurons, and 4 neurons in the output layer. The LBFGS (Limited-memory Broyden–Fletcher–Goldfarb–Shanno) optimizer was chosen, and the logistic function was used as the activation function. The LBFGS optimizer is often used when high convergence speed and good accuracy are required, especially for small- or medium-sized datasets. Its main advantage lies in its ability to converge quickly even on smaller samples and efficient operation with limited memory usage. Graphs illustrating loss functions for the testing and training sets are shown in Figure 7, while predicted versus actual values for each forecast variable along with their corresponding root mean square errors (RMSE) are presented in Figure 8.

The following metrics were used to evaluate the final quality of the developed neural network: RMSE (Root Mean Square Error) for individual measurements, Total RMSE (the overall metric for all parameters), MAPE (Mean Average Percentage Error) for relative error, and R^2^ Score (the coefficient of determination)—a measure of the correspondence between the predicted and true relationships. All obtained metrics are presented in Table 3.

Let’s test the neural network’s performance on new data that wasn’t included in the training and test sets and calculate the percentage error (Table 4).

## 4. Discussion

This study proposes an effective hybrid approach combining neural networks with numerical models for analyzing electromembrane desalination processes, the key mechanism of which is electroconvection. The use of complex variables in neural networks allows for an infinite number of combinations of dimensional quantities. Thus, the use of neural networks allows for the simultaneous analysis of not just isolated individual cases, but entire classes of similar processes, which deepens our understanding of the processes at hand.

A multilayer feedforward neural network was developed, tuned using a randomized hyperparameter search, which improved the efficiency and accuracy of predicting system characteristics. The model achieved the following performance metrics: RMSE = 0.417, MAPE = 3.74% and R^2^ = 0.961. This indicates a strong agreement between the calculated and empirically observed parameters, emphasizing the importance of the proposed approach. The resulting neural network model demonstrated high accuracy in reproducing key process indicators—such as the dimensionless electroconvective vortex diameter, the current–voltage characteristic plateau length, and the onset of electroconvection near the CEM and AEM—based on input parameters including the potential sweep rate, Péclet number, Reynolds number, and $K_{el}$. The application of the proposed method in the future will open up broad prospects for the design of membranes and the development of adaptive strategies for controlling the desalination process.

A key challenge in potentiodynamic modeling is selecting an appropriate potential sweep rate. While a lower rate enhances accuracy, it proportionally increases computational expense. Conversely, a higher rate reduces computation time at the cost of introducing errors due to non-stationary effects. This study identifies a dimensionless sweep rate of 0.07 as the optimal threshold, representing the maximum value for which a quasi-steady-state regime is maintained, thus balancing computational efficiency with model fidelity.

This work represents an important contribution to the development of interdisciplinary research at the intersection of membrane electrochemistry, artificial intelligence and computer modeling.

## Figures and Tables

**Table 1:** Data on the desired characteristics at Re=0.05, Pe=24.39 and $K_{el}=24.74\cdot 10^{5}$ depending on the dimensionless potential sweep rate. Variable d_1_—is the input variable, and variables D_ecv_, L_pl_, ECV_CEM_, ECV_AEM_—are the output variables.

No.	d_1_	D_ecv_	L_pl_	ECV_CEM_	ECV_AEM_
1	0.01	0.342	17.29	25.42	49.19
2	0.04	0.334	17.46	25.5	49.57
3	0.05	0.334	17.54	25.3	48.7
4	0.06	0.35	17.79	25.67	49.22
5	0.07	0.326	17.99	25.77	49.08
6	0.09	0.318	17.62	25.86	50.09
7	0.11	0.346	17.96	26.05	49.58
8	0.12	0.32	17.85	25.83	49.95
9	0.13	0.318	16.85	25.97	49.94
10	0.15	0.32	17.72	26.13	50.21
11	0.16	0.324	17.61	25.85	50.9
12	0.17	0.328	17.64	25.74	48.75
13	0.18	0.33	17.54	26.34	48.29
14	0.19	0.32	17.12	24.13	46.7
15	0.21	0.324	17.7	26.2	48.89
16	0.24	0.35	16.97	25.5	49.28
17	0.39	0.354	7.192	23.35	47.48
18	0.49	0.33	6.97	26.271	49.14

**Table 2:** Training sample. Variable d_1_—is the input variable, and variables D_ecv_, L_pl_, ECV_CEM_, ECV_AEM_—are the output variables. All data in the table are given in dimensionless form.

No.	d_1_	Re	Pe	K_el_	D_ecv_	L_pl_	ECV_CEM_	ECV_AEM_
1	0.01	0.05	24.39	2,470,000	0.342	17.29	25.42	49.19
2	0.01	0.1	48.78	619,000	0.24	15.55	26.77	52.03
3	0.04	0.05	24.39	2,470,000	0.334	17.46	25.5	49.57
4	0.04	0.5	243.9	24,700	0.114	11.44	30.88	56.41
5	0.04	0.1	48.78	619,000	0.22	15.19	25.98	50.63
6	0.06	0.05	24.39	2,470,000	0.35	17.79	25.67	49.22
7	0.06	0.5	243.9	24,700	0.118	11.26	30.53	55.85
8	0.06	1	487.8	6190	0.078	9.87	30.29	60.16
9	0.09	0.05	24.39	2,470,000	0.318	17.62	25.86	50.09
10	0.09	0.5	243.9	24,700	0.124	11.44	30.57	55.74
11	0.09	0.1	48.78	619,000	0.234	15.38	26.37	50.97
12	0.12	0.05	24.39	2,470,000	0.32	17.85	25.83	49.95
13	0.12	1	487.8	6190	0.084	10.14	31.67	61.76
14	0.15	0.05	24.39	2,470,000	0.32	17.72	26.13	50.21
15	0.15	0.5	243.9	24,700	0.11	11.92	31.8	56.19
16	0.15	0.1	48.78	619,000	0.236	15.47	26.56	51.67
17	0.15	1	487.8	6190	0.096	9.89	30.5	58.67
18	0.17	0.05	24.39	2,470,000	0.328	17.64	25.74	48.75
19	0.17	0.1	48.78	619,000	0.23	15.86	26.59	51.82
20	0.17	1	487.8	6190	0.076	9.81	30.17	58.64
21	0.19	0.05	24.39	2,470,000	0.32	17.12	24.13	46.7
22	0.19	0.5	243.9	24,700	0.124	11.12	30.36	56.82
23	0.19	1	487.8	6190	0.08	9.82	30.16	58.77
24	0.24	0.05	24.39	2,470,000	0.35	16.97	25.5	49.28
25	0.24	0.5	243.9	24,700	0.112	11.63	30.89	56.64
26	0.37	0.1	48.78	619,000	0.224	15.46	26.52	51.58
27	0.37	1	487.8	6190	0.08	9.77	30.18	59.08
28	0.49	0.05	24.39	2,470,000	0.33	6.97	26.271	49.14
29	0.49	0.5	243.9	24,700	0.12	11.81	31.62	56.92
30	0.49	0.1	48.78	619,000	0.24	14.9	26.76	50.11
31	0.49	1	487.8	6190	0.086	9.77	29.92	59.35

**Table 3:** Summary metrics.

	RMSE	MAPE	R^2^
D_ecv_	0.020	9.49%	0.960
L_pl_	0.333	2.12%	0.989
ECV_CEM_	0.581	1.99%	0.930
ECV_AEM_	0.736	1.33	0.964
**Total metrics**	**0.417**	**3.74%**	**0.961**

**Table 4:** Verification of neural network performance on hold-out data. Variable d_1_—is the input variable, and variables D_ecv_, L_pl_, ECV_CEM_, ECV_AEM_—are the output variables.

	d1	Re	Pe	K_el_	D_ecv_	L_pl_	ECV_CEM_	ECV_AEM_
Actual Value	0.05	0.05	24.39	2,470,000	0.334	17.54	25.3	48.7
Predicted	0.05	0.05	24.39	2,470,000	0.314	17.4	25.49	49.55
**Error in %**	-	-	-	-	**6**	**0.8**	**0.8**	**1.8**
Actual Value	0.37	0.05	24.39	2,470,000	0.352	14.23	25.2	48.64
Predicted	0.37	0.05	24.39	2,470,000	0.338	13.8	25.38	49.04
**Error in %**	-	-	-	-	**4**	**3.1**	**0.8**	**0.8**

## Data Availability

The authors confirm that the data will be made available upon request.

## Abbreviations

The following abbreviations are used in this manuscript:

CEM	Cation-exchange membrane
AEM	Anion-exchange membrane
ECV	Electroconvective vortex
EMS	Electromembrane systems
DC	Desalination channel
CVC	Current–voltage characteristic
ANN	Artificial neural networks
AI	Artificial intelligence
